# A New, Quick, and Simple Protocol to Evaluate Microalgae Polysaccharide Composition

**DOI:** 10.3390/md19020101

**Published:** 2021-02-10

**Authors:** Antoine Decamp, Orane Michelo, Christelle Rabbat, Céline Laroche, Dominique Grizeau, Jérémy Pruvost, Olivier Gonçalves

**Affiliations:** 1 Université de Nantes, GEPEA, UMR CNRS 6144, 37 boulevard de l’Université, 44600 Saint-Nazaire, France; antoine.decamp@etu.univ-nantes.fr (A.D.); orane.michelo@etu.univ-nantes.fr (O.M.); Christelle.rabbat@imt-atlantique.fr (C.R.); Dominique.Grizeau@univ-nantes.fr (D.G.); jeremy.pruvost@univ-nantes.fr (J.P.); 2 Institut Pascal UMR 6602, Université Clermont Auvergne, CNRS, SIGMA Clermont, 63000 Clermont-Ferrand, France; celine.laroche@uca.fr

**Keywords:** enzymatic quantification, rapid and cost-effective method, easy-to-use bioactive (exo)polysaccharide profiling, microalgae

## Abstract

In this work, a new methodological approach, relying on the high specificity of enzymes in a complex mixture, was developed to estimate the composition of bioactive polysaccharides produced by microalgae, directly in algal cultures. The objective was to set up a protocol to target oligomers commonly known to be associated with exopolysaccharides’ (EPS) nutraceutical and pharmaceutical activities (i.e., rhamnose, fucose, acidic sugars, etc.) without the constraints classically associated with chromatographic methods, while maintaining a resolution sufficiently high to enable their monitoring in the culture system. Determination of the monosaccharide content required the application of acid hydrolysis (2 M trifluoroacetic acid) followed by NaOH (2 M) neutralization. Quantification was then carried out directly on the fresh hydrolysate using enzyme kits corresponding to the main monosaccharides in a pre-determined composition of the polysaccharides under analysis. Initial results showed that the enzymes were not sensitive to the presence of TFA and NaOH, so the methodology could be carried out on fresh hydrolysate. The limits of quantification of the method were estimated as being in the order of the log of nanograms of monosaccharides per well, thus positioning it among the chromatographic methods in terms of analytical performance. A comparative analysis of the results obtained by the enzymatic method with a reference method (high-performance anion-exchange chromatography) confirmed good recovery rates, thus validating the closeness of the protocol. Finally, analyses of raw culture media were carried out and compared to the results obtained in miliQ water; no differences were observed. The new approach is a quick, functional analysis method allowing routine monitoring of the quality of bioactive polysaccharides in algal cultures grown in photobioreactors.

## 1. Introduction

Polysaccharides can be synthetized by different organisms (bacteria, fungi, macroalgae, microalgae, terrestrial plants, etc.). Widely recognized as polymeric carbohydrate molecules, polysaccharides consist of elongated linear or branched chains of monosaccharides (rhamnose, fucose, galactose, glucose, xylose, uronic acids, etc.) linked by glycosidic bonds. These macromolecules exist in two forms: homopolysaccharides, which comprise just one type of monosaccharide, and heteropolysaccharides, which comprise two or more types of monomer units. In the specific case of microalgae, polysaccharides may be intracellular, which are accumulated inside algal cells and known as storage polysaccharides, or extracellular, which are directly bonded to the microalgae cell membrane as bound polysaccharides (BPS) or released into the culture environment as exopolysaccharides (EPS). Polysaccharides produced by microalgae have robust biological functions depending on the composition of the polysaccharide, which, in turn, depends on the culture conditions and the taxonomy of the algae [1]. Until now, these biomolecules have been principally studied for their physicochemical properties, proposing therefore potential new hydrocolloid agents because of their thickening [2] and gelling properties [3]. In addition, most cyanobacteria exopolysaccharides (EPS) have adhesive properties that could also find application in the environmental industry for aggregating soil and sand particles to avoid sediment erosion [4].

Polysaccharides act as antioxidant, anticancer [5], antibacterial, antiviral, hypercholesteremic [6], anti-inflammatory [7,8,9,10], or anti-parasitic [8] agents, and there is extensive interest in their biological properties and potential applications in nutraceutical and pharmaceutical sectors. These pharmaceutical properties are governed by the EPS composition (associated with the presence of rare oligosaccharides such as rhamnose or fucose, for example), net charge (associated with the presence of sulfate groups or acidic monosaccharides), and size and chain conformation in space [5,9,10,11]. Microalgae are organisms with a high metabolic plasticity, which enables them to adapt their physiology to different types of environments. Changing the culture conditions can trigger the overproduction of polysaccharides, as is the case with nutrient limitation, but can also induce strong changes in the composition of the polymer, modulating therefore its bioactivity. For example, an increase in salinity leads to a decrease in the xylose content of EPS produced by the red microalga *Porphyridium cruentum* from 54% to 38% of the total osidic content [12]. An increase in the net charge of the EPS of *Porphyridium cruentum* (sulfate and acidic monosaccharides) would, on the contrary, improve their antitumoral and antiviral activities [13]. Other parameters such as the light intensity for *Arthrospira platensis* (decrease in the amount of rhamnose and acidic monosaccharides and increase in the galactose content for high irradiance [14]) or the wavelength of the light energy source for *Nostoc flagelliforme* may induce changes in their EPS’s monosaccharide composition, modulating therefore their potential bioactivities (for example, cultivation with yellow light causes an increase in the amount of fructose and a decrease in the amount of galactose [15]). The need for monitoring the polysaccharide (PS) composition as easily as it could be appears mandatory if one wants to valorize those macromolecules, since it allows one to guarantee the repeatability of the produced PS in terms both of quantity and quality (constant composition for constant biological activities).

Over the past few years, different methodologies have been developed for determining the PS monomeric composition. Gas chromatography (GC) is commonly used, but this method requires derivatization to enable the volatilization of simple sugars [16]. Gas chromatography offers high-quality analysis of the composition of monosaccharides, especially when carried out in conjunction with mass spectrometry (GC-MS), thus providing better resolution and sensitivity [17]. Although mass spectrometry enables carbohydrates to be easily distinguished from other organic compounds, quantification through a flame-ionization detector (FID) is used more frequently to measure the amount of derivatized monosaccharides due to its high reproducibility [18]. However, to quantify monosaccharides, it must be ensured that the derivatization is complete in order to avoid loss or degradation of the analyzed sugars. In addition, some monosaccharides such as uronic acids are difficult to derivatize, which in some cases prevents accurate quantification with gas chromatography. Another drawback to this method is that the same monomer may present different derivatized forms and consequently present several spectra for the same molecule, thus complicating the analysis [3].

The monosaccharide composition of microalgal EPS can also be determined by high-pressure liquid chromatography (HPLC) using a refractive index detector [19]. This saves time in preparing the samples compared to GC since no derivatization step is required. HPLC separates the molecules according to their electrical charge and polarity. However, in terms of monosaccharide composition, polarity is the main mechanism that enables separation, and this method compromises the chromatographic resolution, resulting in biases when dealing with complex mixtures [17,20]. For example, two different compounds (xylose and galactose) presenting similar polarities or structure may therefore be co-eluted.

Another method, high-performance anion-exchange chromatography (HPAEC) with pulsed amperometric detection (PAD) can also be used to determine the monosaccharide composition of microalgae EPS [8,21]. This method provides high-resolution analysis for the most commonly occurring monosaccharides, with no need for the derivatization step. Monosaccharides are separated according to the differences in pKa. However, the retention time and elution order may be strongly affected by any changes in the temperature or alkalinity of the mobile phase. The signal may also be lost in the event of inadequate and/or insufficient flushes of the column, and the electrochemical response may decrease if proteins and peptides are found in the sample [22].

Different methods based on the use of nuclear magnetic resonance (NMR) systems have been developed to quantify or to profile polysaccharides. Studies have shown that the use of ^1^H NMR, when preceded by the Saeman hydrolysis procedure [23], could identify and quantify the osidic monomer composition of the main food polysaccharides (high acyl gellan, xanthan, pectins, and locust bean gum) [24]. With other NMR approaches based on the chemical-shift-selective filtration with total correlation spectroscopy (CSSF-TOCSY) sequence, 22 different saccharides can be distinguished simultaneously in an aqueous medium without pre-treatment, even if they are in the form of a monomer (glucose), dimer (sucrose), or trimer (raffinose). These methods, although fast, sensitive, and efficient, are nonetheless quite expensive and complex since they require a wide range of analytical devices and very experienced people [25].

Despite having numerous advantages, these methods also present limitations as they are slow, sample consuming, and costly. They generally require pre-treatment or pre-purification of the sample by diafiltration for salt removal, as low sample purity prevents accurate quantification. To rapidly follow changes in the EPS composition during microalgae cultures, it would therefore be useful to develop a quick, simple, cheap, sample-saving, and specific quantification method to estimate the composition of the microalgae EPS and therefore ensure repeatability of the produced bioactive polysaccharides. It could allow one to easily and efficiently follow the EPS composition over time, obtaining essential information about the metabolic plasticity of the EPS biosynthesis occurring in microalgae and allowing one to gain mastery over this information for future production processes for nutraceutical or pharmaceutical applications. In this work, an original method complementary to the classical chromatographic approaches is developed for quick and easy profiling of EPS, targeting, especially, monosaccharides commonly associated with nutraceutical or pharmaceutical activities (fucose, rhamnose, glucuronic/galacturonic acids). It consists, with prior knowledge of the major sugar in the EPS composition, of choosing ad hoc enzymes to quantify each targeted monomer. The enzymes should bring sensitivity and specificity to the protocol because of their intrinsic properties, and the micronization of the assays would significantly reduce the cost of the profiling approach. The results and perspectives emerging from the evaluation of the profiling ability of commercial kits are presented hereafter for the EPS produced by three different marine microorganisms belonging to a genus with strong applicative potential in the nutraceutical or pharmaceutical sector. One of them is already well known, *Porphyridium cruentum*, and should be considered a model strain for this study. The other two are totally new and come from a screening procedure whose potential has been assessed in [1].

## 2. Results and Discussion

Hereafter, the development and validation of a method allowing easy profiling of the constitutive monosaccharides of microalgae exopolysaccharides is proposed. This method can be used once the sugar profile is determined with a reference analytical technique (HPLC, GC, NMR etc.). Prior knowledge about the sugar profile is necessary for following the present approach, since it determines the choice of the enzymes to be employed for the targeted quantification of the different monomers identified (i.e., an L-fucose deshydrogenase for the fucose, an L-rhamnose dehydrogenase for the rhamnose, etc.). The present approach exploits enzyme sensitivities and specificity properties that are directly used at their maximum in the complex environment of both sample pre-treatment and microalgae production. The usage of microtiter plates permits the multiplexing and micronization of the technique, allowing the method to be quick, easy to use, and cost saving. The performances and limit of usage of the method are assessed in the following sections.

### 2.1. Analytical Performance of the Method

The aim of this part was to carry out an assay of the studied monosaccharides for different concentrations (ranging from 8.26 × 10^−5^ to 0.21 g·L^−1^ and measured in quintuplicate) to determine the limits of detection, quantification, and saturation for each enzymatic kit and to compare them with existing methods. The evolution of absorbance at 340 nm was measured for different concentrations. Each measurement was carried out in quintuplicate, and the values of the coefficients of variation were calculated. This revealed the minimum concentrations to be studied in order to obtain coefficients of variation of less than 10%, while remaining within the limit of linearity of the method. These experimental results were used to calculate the LOD and the LOQ according to Equations (1) and (2) [26]. The limits of quantification for the enzymatic assays were identified as being on the order of 1 log. These values appeared to be low enough to consider this technique as being competitive with the reference chromatographic techniques (Table 1).

In Table 1, strong heterogeneity could be observed in the distribution of the limits of quantification, ranging from about one-hundredth log to one hundred log of nanograms by injection. It seemed to depend on the used method but also on the studied monosaccharides. According to this criterion, the most performant methods were LC-MS-MS and the RP-HPLC (1), where the limits of quantification were calculated as being on the order of one-hundredth of log. HPAEC UV-VIS and the RP-HPLC (2) were identified as being slightly less sensitive, with limits of quantification on the order of one-tenth of log. The enzymatic method was presented here as an alternative method with a log-order limit of quantification that positions it in a similar range of sensitivity as that of chromatographic methods such as the borate-HPAEC, GC-FID (1), GC-MS, and the LC-MS. However, it presented very interesting performances for the quantification of the monosaccharides commonly associated with EPS nutraceutical or pharmaceutical bioactivities (fucose, rhamnose, glucuronic/galacturonic acids). There were also less performing chromatographic techniques with higher limits of quantification, such as GC-FID (2) and TLC, where the limits of quantification were on the order of one thousand of log. It would have been interesting to study the limits of quantification at the polymer scale (g monosaccharide/100 g polysaccharides) as developed for NMR methods for polysaccharides [24] and food matrices [25].

### 2.2. Accuracy of the Method underExtreme EPS Hydrolysis Conditions

This part aims at verifying whether the acidification-neutralization sequence (using TFA and NaOH) followed during the EPS hydrolysis pre-treatment protocol did not inhibit the activities of the enzymes used for the quantification of the released monomers. For that purpose, the experiment was conducted with an artificial sugar mixture of known concentration and representative of the polysaccharide composition of *Pavlova* sp. The activities of the enzymes were measured in the presence of 1 M TFA and NaOH.

**Figure 1:**
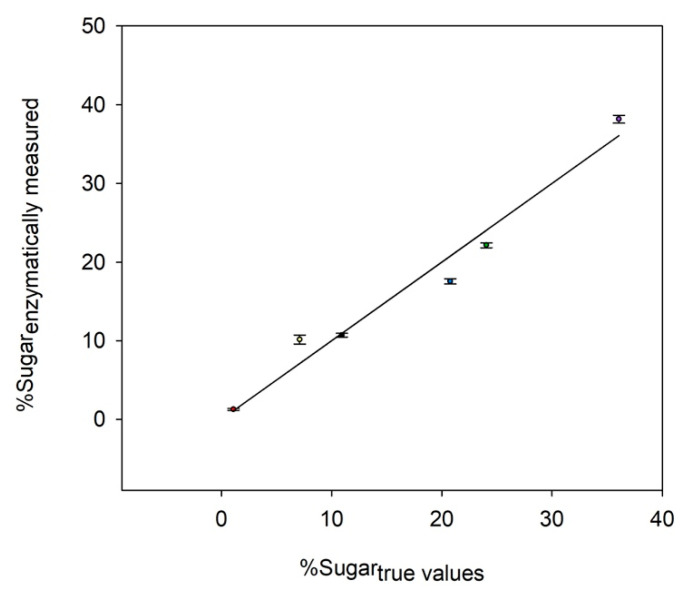
Accuracy of the enzymatic method after the acidification-neutralization sequence protocol (TFA and NaOH). The scatter plot illustrates the correlation between the enzymatic measurements and the sugar true values expressed in dry weight ratio (%). The error bars correspond to the average of 5 independent measurements for fucose (red dot), uronic acids (yellow dot), xylose (black dot), glucose (blue dot), galactose (green dot), and rhamnose (purple dot). The value of the average coefficient of variation was 4% for all the considered measurements. The first bissectrice is drawn in black. Above this line, the enzymatic measured values are over-estimated; under the line, they are under-estimated.

Figure 1 illustrates the comparison between the enzymatic method and each monosaccharide’s true value. A good agreement was obtained between the relative amount quantified by the enzymatic assay and the true concentration of each sugar. Only a slight error was identified for the quantification of neutral sugars (i.e., glucose, galactose, fucose, and xylose), with a deviation from the true values ranging from 2% to 18%. For glucuronic acids, they all seemed to be over-estimated at 30% from the true value, so data should be carefully interpreted when observing the modulation of these monosaccharides on real samples. Despite the latest result, data indicated that the enzymes were still operational in the presence of TFA and NaOH in the medium, thus validating their usage directly onto hydrolyzed samples after neutralization, and that without an additional step. The enzymatic profiling method could be therefore considered as being able to accurately measure the glucosidic profile of an artificial mixture.

### 2.3. Kinetics of Hydrolysis of the EPS Glycosidic Linkages

**Figure 2:**
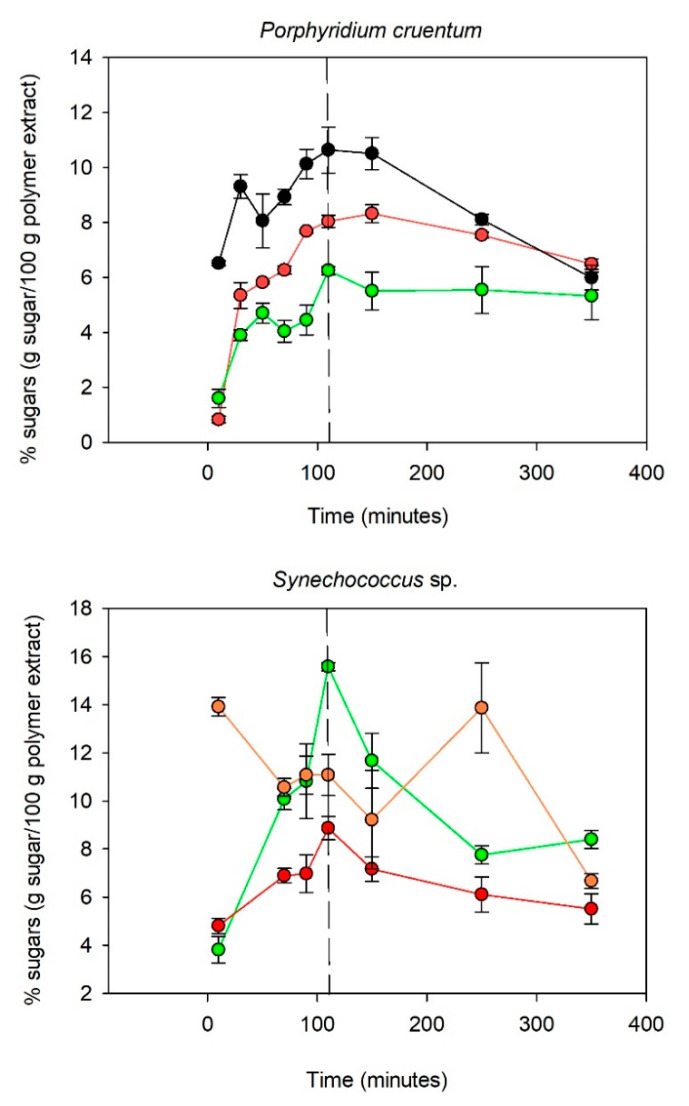

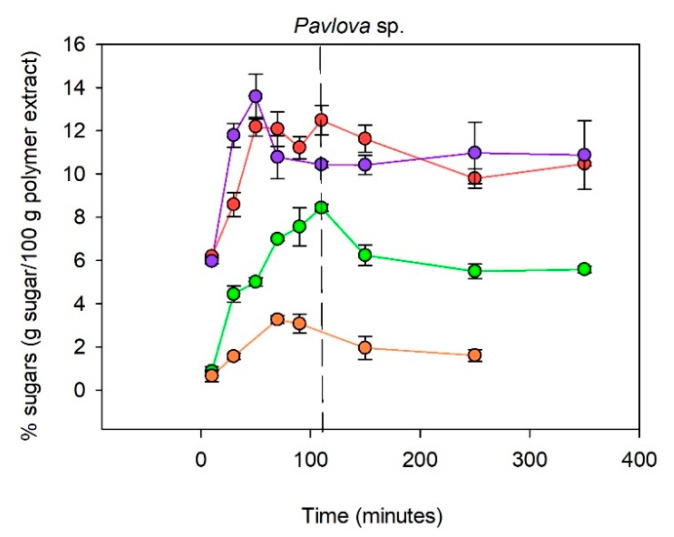
Hydrolysis kinetics of the polysaccharides produced by *Porphyridium cruentum*, *Pavlova* sp., and *Synechococcus* sp. (red = galactose; black = xylose; green = glucose; orange = fucose; purple: rhamnose). The error bars correspond to the average of 5 independent measurements.

Prior to any analysis of a polysaccharide’s composition, it is mandatory to cleave the glycosidic linkages of the polymers in order to release their constitutive monosaccharides. One of the most used methods requires hydrolysis by a mineral acid such as sulfuric, chlorohydric, or trifluoroacetic acid. Various conditions of hydrolysis have already been reported, and TFA usage (2 to 4 M, 90 to 240 min, 100 to 120 °C) has demonstrated optimal performances [16]. However, it is noteworthy that degradation of the released monosaccharides has been reported as a major difficulty in their analysis, especially when drastic conditions are needed to ensure the breakdown of the more resistant glycosidic bonds [34]. Therefore, it was mandatory to determine an optimal hydrolysis time for the present approach. This was performed by studying the kinetics of the hydrolysis of the EPS of our three model strains (i.e., *Pavlova* sp., *Porphyridium cruentum*, and *Synechococcus* sp.) and quantifying over time their constitutive monosaccharides. The aim of these kinetics experiments was to determine an optimal condition applicable for all samples, allowing one to find a compromise between (i) complete hydrolysis of the EPS and (ii) minimization of monosaccharide degradation. The obtained results are presented in Figure 2.

Figure 2 indicates that the kinetics of hydrolysis were polymer dependent. For *Porphyridium cruentum*, the polysaccharide was mainly composed of galactose, glucose, and xylose (90% of the polymer). The monosaccharides appeared to be totally released after 110 min of hydrolysis time. After 110 min, the xylose content decreased sharply, while the galactose and glucose content diminished only slightly. Xylose degradation is known to occur faster mainly because of its chemical structure since pentoses are less stable than hexoses [35]. Similar hydrolysis kinetics were obtained for the *Synechococcus* sp. polymer regarding glucose and galactose liberation. The fucose content appeared to be more fluctuant over the time. A maximum appeared after 10 min of hydrolysis and decreased slightly up to 110 min. However, an increase in the fucose content was observed between 150 and 250 min. Unlike with *Porphyridium cruentum*, the glucose content decreased sharply after 110 min of hydrolysis, which could be explained by a higher protein content in the *Synechococcus* sp. extract than in the *Porphyridium cruentum* extract. Since acid hydrolysis is a reaction that takes place at 120 °C, the presence of proteins in addition to carbohydrates could favor the Maillard reaction. The Maillard reaction is a condensation reaction involving an amino acid and a reducing sugar, causing the degradation of the latter through the formation of secondary products such as Amadori compounds (aldosamines, cetosamines), dehydroreductones, hydroxymethylfurfural, and melanoidins [36]. The hydrolysis of *Pavlova* sp. polysaccharides released a maximum amount of glucose and galactose after 110 min, whereas for rhamnose and fucose, the highest amounts released in the medium were, respectively, after 50 and 90 min of acidic treatment.

These experiments confirmed that monosaccharide release (and degradation) varies with the nature of the sugar (ketose, aldose, or uronic acids [3]) but also depends on the spatial configuration of the polysaccharide. This hypothesis could explain why the release and degradation rates of a given monosaccharide were different between the studied samples. For instance, fucose release was faster for the *Synechococcus* sp. PS than for the *Pavlova* sp. PS. Moreover, the fluctuation observed in the fucose concentration from the *Synechococcus* sample might also be explained by the structural conformation of the polysaccharide. During the depolymerization process, the polymer expands, revealing new hydrolysis sites, potentially more fucose-rich and consequently leading to late release. This could also be linked to a difference in molecular mass, viscosity, or conformation of the polysaccharide, which could change the accessibility of the hydrolysis sites.

A study of the degradation kinetics of the polymers during acid hydrolysis was essential because it enabled the identification of an optimal consensus time for the release of a maximum amount of free monomers (for a given temperature and TFA concentration). For *Porphyridium cruentum*, the optimal time was 110 min for all tested monomers. As with *Porphyridium cruentum*, the highest amounts of galactose and glucose obtained from *Synechococcus* sp. and *Pavlova* sp. EPS were after 110 min of hydrolysis. However, fucose and rhamnose degraded more rapidly, and their content decreased by 20% after 110 min of hydrolysis (compared to the maximum amount), so specific attention should be paid when targeting these essential monosaccharides for the monitoring of bioactive EPS. Ideally, the optimal degradation time should be adapted to the composition of the studied polymer (if it contains heat-sensitive sugars of pharmaceutical interest). However, due to standardization constraints, it appeared necessary to choose a common hydrolysis time for all the studied samples. The optimal time, in the case of *Synechococcus* sp. and *Porphyridium cruentum*, was around 110 min compared to 70 min for the polysaccharides synthesized by *Pavlova* sp. However, the degradation of monosaccharides from the *Pavlova* sp. polymer significantly reduced between 70 and 110 min, so the hydrolysis time of 110 min was retained.

### 2.4. Estimation of Potential Interferences of Maillard Compounds toward the Method

The Maillard reaction induced the formation of several compounds (i.e., melanoidins), with some of them presenting absorption properties close to the wavelength range used by colorimetric assays. Since enzymatic quantification of carbohydrates involves the use of spectrophotometry, it appeared mandatory to evaluate the potential interference of Maillard reaction products with it. Another source of bias associated with Maillard reaction product formation should also be taken into account. Since some of these compounds (i.e., Amadori products) are structurally analogous to the targeted monosaccharides, they could strongly affect the sensitivity of the enzymatic kits by acting as competitive inhibitors. To verify this hypothesis, we proposed to artificially increase the amount of the principal substrate (i.e., glucose after the hydrolysis step) to augment the formation of Amadori products and assess whether a bias could be associated with it. Therefore, 5 µL of a 2 g·L^−1^ glucose solution was added to 245 µL of hydrolysate obtained from the hydrolysis of *Pavlova* sp. PS, increasing the concentration by 0.04 g·L^−1^ of glucose. No significant alteration of measurement could be observed, since this standard addition test led to a 97% recovery rate, suggesting that Maillard reaction products should not interact with the enzymes employed for the quantification. The present method therefore appeared to be robust enough regarding the potential formation of Maillard reactions products.

### 2.5. Accuracy of the Method in Real Conditions

**Figure 3:**
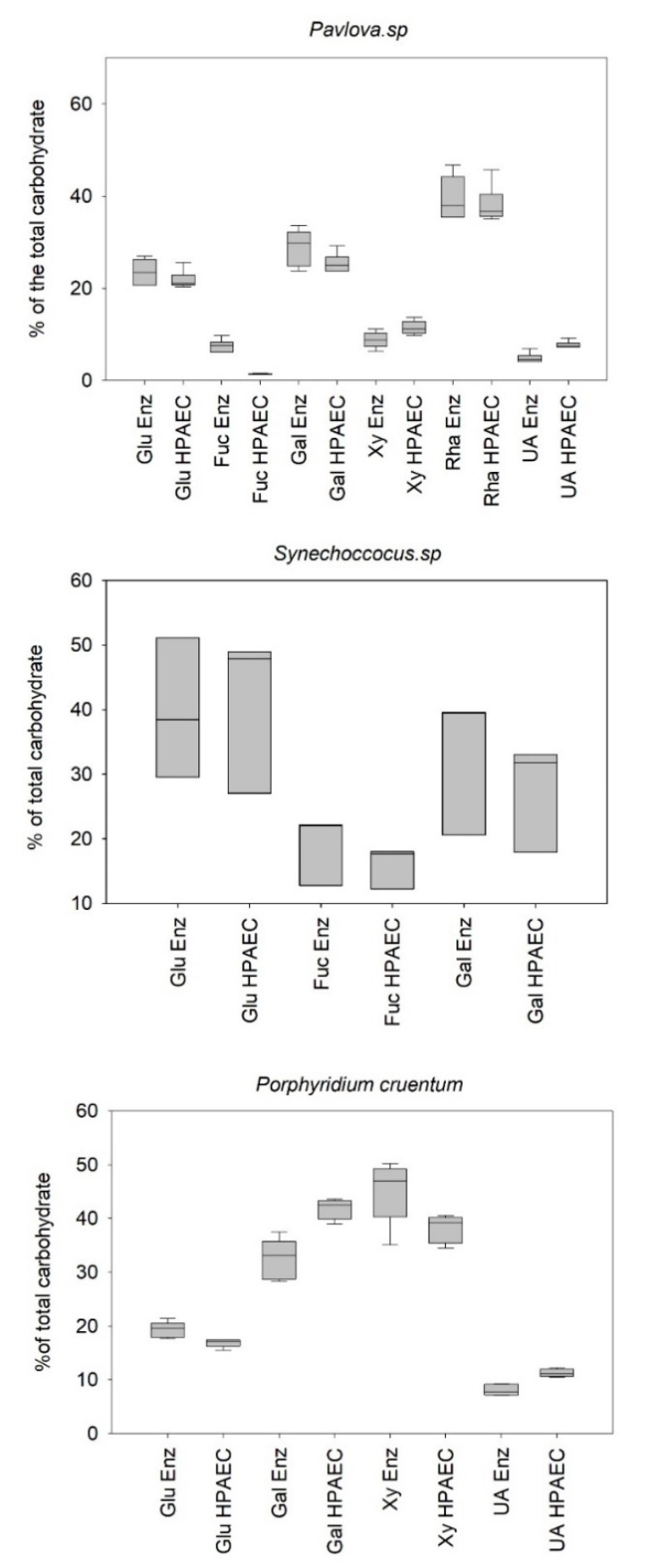
Comparison between the profile determined by HPAEC-PAD and the enzymatic approach for three different EPS. Results are given as box plots, e.g., the boundary of the box closest to zero indicates the 25th percentile, the line within the box marks the median, and the boundary of the box furthest from zero indicates the 75th percentile. Whiskers (error bars) above and below the box indicate the 90th and 10th percentiles (measurement replicates: 5; hydrolysis replicates: 5 for *Porphyridium cruentum* and *Pavlova* sp. and 3 for *Synechococcus* sp.). (Xy: xylose; Glu: glucose; Gal: galactose; UA: uronic acids; Fuc: fucose; Rha: rhamnose; Enz: enzymatic).

The previous results suggested that the enzymes used in this approach were not affected by the presence of Amadori products or the TFA/NaOH mixture, conserving their specificity. The results also suggested that the present method could be used straightforwardly in real conditions since the developed protocol was reliable enough. Therefore, it was assessed on the EPS of the three model strains to quantify each essential monosaccharide (i.e., representing more than 90% of the microalgae polysaccharide composition). The enzymatic results were compared to the HPAEC-PAD method, chosen as a reference method for monosaccharide quantification. EPS were hydrolyzed (five replicates for *Porphyridium cruentum* and *Pavlova* sp. and three replicates for *Synechoccocus* sp.) depending on the selected protocol. Following NaOH neutralization, the monomers of each hydrolysate were then quantified in quintuplicate to obtain results, combining the variability associated with the hydrolysis and to the measurement method. The results were expressed in percentages of each monomer for a given PS sample for standardization purposes. Figure 3 illustrates the results obtained from the analysis of monosaccharide profiles using the reference method (HPAEC) and the enzymatic method. Table 2 and Table 3 show the errors between the developed methodology and the reference method and their corresponding variability.

Concerning, the quantification of the *Pavlova* sp. monosaccharide extract using enzymatic and reference methods, no significant difference in glucose, galactose, and rhamnose percentage determination could be assessed (*p*-value > 0.05) (Table 2). However, a significant difference was observed in the determination of the percentages of xylose, uronic acids, and fucose. Despite these statistically significant differences, the results showed an error of less than 20% for xylose, unlike fucose and uronic acids, with errors of 82% and 35%, respectively. Here again, it will be important to pay specific attention when targeting these essential monosaccharides associated with the bioactivity of EPS. Concerning the intrinsic distribution of the error (Table 3), it can be seen that the average intrinsic error was estimated at approx. 9.8% for the reference method compared to approx. 15.2% for the enzymatic method, which makes the reference method a more repeatable method. The overall measurement error of the profile was calculated by standardizing the error observed for each monomer with the abundance of the given monomer. The results showed that the profile determined for the extract produced by *Pavlova* sp. was 83%, coinciding with the reference profile obtained by HPAEC.

Concerning the determination of the composition of the polymer obtained from *Synechococcus* sp. extract by both methods, no statistically significant differences could be assessed for galactose, glucose, and even fucose. Unlike the results obtained when studying the extract produced by *Pavlova* sp., the fucose content coincided with the results obtained by the reference method (Table 3). In addition, the intrinsic errors (Table 4) revealed a percentage of variability identical for the two methods and almost twice as that estimated for the two others strains. It suggested a strong difference between the studied aliquots of PS that could probably be associated with a heterogeneous distribution of the polymer in the purified extract. Nevertheless, the overall error calculated for the polysaccharide compositions produced by *Synechococcus* sp. was still acceptable (c.a. 12%), letting us consider the enzymatic methodology to reliably analyze the monosaccharide profiles as determined by the reference method.

Considering the profiling of the polymer produced by *Porphyridium cruentum,* significant differences for all the monosaccharides studied could be observed (*p*-value < 0.05) (Table 3), with a global deviation from the reference method almost twice of that observed for the two other strains. The quantification of galactose was particularly impacted. The polymer secreted by *Porphyridium cruentum* contains l- and d-galactose [37,38] as does the polymer secreted by *Porphyridium* sp. [39,40]. Unlike the reference method, which quantifies indifferently the L and D forms of galactose, the enzymatic method quantifies specifically the D isomer, leaving the L form unquantified, therefore underestimating the total amount of free galactose. This hypothesis may partially explain the difference observed in the galactose quantification for the EPS from red microalga. Nevertheless, the percentage of error obtained for the complete determination of the profile of this polysaccharide was still acceptable (c.a. 22%).

### 2.6. Robustness of the Method in Production Conditions: Direct Application on Growth Medium

**Figure 4:**
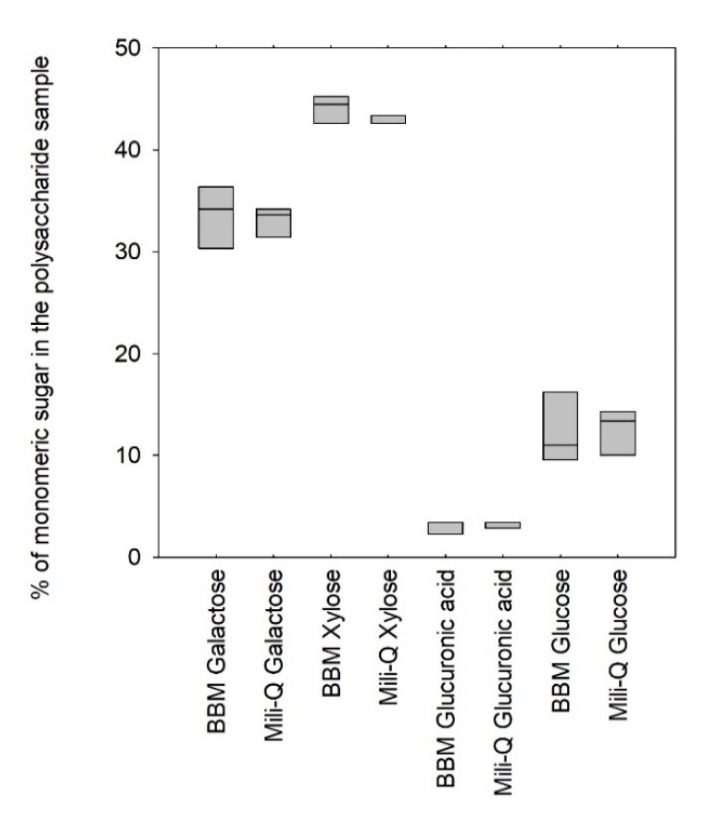
Profile comparison for EPS diluted in Bold’s basal medium (BBM) culture medium and milli-Q water prior to hydrolysis.

All the experiments conducted so far have suggested that the methodology for the determination of the enzymatic profile of the polysaccharides synthetized by microalgae is sufficiently specific and sensitive to be used as a monitoring approach for the evolution of the compositional quality of EPS in a complex environment (i.e., in the presence or absence of TFA and NaOH (Section 3.2) as well as in a hydrolysate containing Maillard reaction products (Section 3.4)). In addition, the comparison with a reference method enabled us to confirm the robustness of the method with different types of microalgae polymers. With the objective of using the enzymatic method as a quick routine procedure to determine the EPS composition in the time course of algae cultivation, it also appeared important to verify whether the salts of the culture medium could interfere. Chromatographic methods require sample purification before analysis, and the polymers tested in the previous experiments were all diafiltered prior to hydrolysis. These steps are time consuming and involve an additional risk of material loss. Figure 4 shows the results of the profiling of the polymers produced by the *Porphyridium cruentum* strain when diluted in Bold’s basal medium (BBM) and mili-Q water.

Figure 4 indicated that no significant difference could be assessed when profiling the polysaccharide produced by *Porphyridium cruentum* in pure water or in typical BBM culture medium, suggesting a negligible influence of the salts on enzyme activities (*p*-value > 0.05). These results were of first importance since they demonstrated that the EPS-profiling method could be applied directly on culture supernatants and is therefore suitable for use as a routine fast method for monitoring the EPS production in photobioreactors.

### 2.7. Comparison of the Enzymatic Profiling Method with a Classic Chromatography Approach (i.e., HPAEC)

Table 4 proposes a simplified summary of the strengths and weaknesses of the two methods used for profiling the polysaccharides studied.

First, it is important to note that a chromatographic method requires more time than the enzymatic method (92 min per injection for the HPAEC method). As each monosaccharide is encountered in various amounts in the polymer, analysis is generally needed to be performed on three different dilutions to obtain peaks with areas in the range of the standard curves for all the monomers, thus requiring 276 min. Additionally, the calibration requires injection of five concentrations of a standard monosaccharide mix, increasing the analysis time to 736 min. In the case of enzymatic detection, around 30 min per monosaccharide is needed. The use of a chromatographic technique for monitoring monosaccharide profiles also requires a suitable chromatography system, column, and detector. In addition, working with high-resolution systems requires more frequent maintenance. This is not the case with the present enzymatic monitoring method, which requires less investment. Only the acquisition of appropriate kits (~€208/kit (550–1150 assays)), microplates, and a microplate reader is needed. The water consumption is also reduced for the enzymatic approach when compared to the reference method, because of the micronization of the microplate reader system.

### 2.8. Discussion 

The profiling of microalgae polysaccharides with an enzymatic approach appears to be a useful method since it enables the quick and easy constitutive monosaccharide determination to be monitored, with no need to invest in expensive chromatography equipment. Even if it requires prior knowledge of the major sugars’ profiles in order to determine the appropriate enzyme choice, it can be performed by using an external analytical service, especially devoted to it. In addition, it is important to note that contrary to gas chromatography systems, the usage of enzymatic kits does not require derivation nor diafiltration (to remove interfering salts), thus limiting material loss and making significant savings in terms of sample preparation time. However, as the polysaccharide concentration in the microalgae culture medium is somehow not abundant enough (between 0.1 and 0.6 g·L^−1^, depending on culture conditions), a concentration step should be envisaged.

The method is also cost and time saving. For the EPS of *Pavlova* sp., the determination of the profile required six kits (€1250), each kit allowing an average of 1000 assays. It corresponded to an approximate price of €3.64 per profile realized in monoplicate (€9.72 if calibration curves are included). The total monosaccharide profile of the EPS produced by *Pavlova* sp. could be obtained in less than 2 h, which was very interesting since it adapted to the biological timescale response of microalgae, which is the order of the day.

Finally, the main properties of enzymes (i.e., their sensitivity and specificity) are exploited here at their maximum potential in a very complex mixture and without being affected by the presence of interfering substances. The method is robust enough to be used directly in the complex environment of both sample pre-treatment and microalgae production, allowing quick and easy monitoring of EPS quality during the production process. For absolute quantification of monosaccharides commonly associated with EPS’s nutraceutical and pharmaceutical activities, specific attention is required. When targeting rhamnose, fucose, and uronic acids, errors could reach values close to 11%, 82%, and 35%, respectively. Absolute quantification results for rhamnose and uronic acids remained very acceptable, whereas those for fucose suggested careful interpretation and cross-validation using other chromatographic techniques. Fucose appeared to be much more labile than the other sugars, and specific soft conditions should be systematically applied for the depolymerization of fucose-rich EPS. It would be also interesting to evaluate the limits of quantification of monosaccharides at the polymer scale (g monosaccharides/100 g polysaccharides) under production conditions and to compare the obtained results with a crude culture supernatant, a concentrated supernatant, and a diafiltered-concentrated supernatant.

## 3. Material and Methods

### 3.1. Strains, EPS Production, and Purification

Development of the new methodology required cultivation of three EPS producers: *Pavlova* sp. (RCC 3438), *Porphyridium cruentum* (Utex 161: https://utex.org/ (accessed on 6 January 2021)), and *Synechococcus* sp. (RCC 2380) (http://roscoff-culture-collection.org/ (accessed on 6 January 2021)).

The different microalgae were cultivated in a 1 L flat-panel airlift photobioreactor in Bold’s basal medium (BBM) with the following nutrient concentrations (g·L^−1^): 4.5 NaNO_3_, 0.45 MgSO_4_·7H_2_O, 0.05 CaCl_2_·2H_2_O, 0.05 EDTANa_2_·2H_2_O, 0.028 FeSO_4_·7H_2_O, 0.45 K_2_HPO^4^, 0.369 KH_2_PO_4_, 1.26 NaHCO_3_, 4 × 10^−4^ ZnSO_4_·7H_2_O, 8.8 × 10^−5^ Co (NO3)2·6H_2_O, 1.58 × 10^−4^ CuSO_4_, 5.8 × 10^−3^ H_3_BO_3_, 3.6 × 10^−3^ MnCl_2_·4H_2_O, and NaCl 15 g·L^−1^ for *Porphyridium cruentum* and 28 g·L^−1^ for *Pavlova* sp. and *Synechococcus* sp. Mixing was by air injection, and the pH was set at 7.5 by pure CO_2_ injection. The temperature was maintained by air-conditioning (22 °C inside the photobioreactor). The photon flux density (PFD) was supplied by white light-emitting diode (or LEDs) (Bio-concept technologies, Paris, France) and fixed at 90 µmol·m^−2^·s^−1^ for the whole cultivation time (20 days).

Isolation of soluble EPS in the culture media was carried out as follows, mainly to remove the salts present in the culture broth in order to perform proper estimation of the total polysaccharide amount using the method described in [1]. The biomass was first harvested by centrifugation at 4420× *g* for 10 min (Mikro 22 R, Hettich, Germany). The salts contained in the supernatant were removed by a concentration and diafiltration process using a sartorius filtration pilot (Sartojet, Sartorius, Germany) and a 50 kDa organic membrane (final conductivity 50 µS·cm^−1^). Finally, all the retentates were pooled and lyophilized using freeze-dryer Alpha 1-2 LD plus (Christ, Germany) for 24 h of primary drying (1 mbar) and 48 h of secondary drying (0.012 mbar). Each pooled EPS fraction was then quantified in terms of total carbohydrates using the approach described in [1] in order to estimate their purity in the final lyophilizate (35% dry weight (or DW) for *Synechococcus*, 45% DW for *Porphyridium*, and 62% DW for *Pavlova*). Calculations systematically took into account these purity values for all subsequent experiments.

### 3.2. Hydrolysis of Native EPS

Prior to any composition analysis, acid hydrolysis of EPS was mandatory to break glycosidic bonds and release monosaccharides. Hydrolysis was carried out on 15 mg of polysaccharides in 2 mL of 2 M trifluoroacetic acid (>99%, Acros Organics, Geel, Belgium) at 120 °C (heat block; SBH130D Stuart Scientific, London, UK) for 110 min in 8 mL glass vials. During hydrolysis, the samples were mixed using a vortex to avoid aggregation. After hydrolysis, the TFA (2 M) was neutralized by adding 2 mL of NaOH (2 M).

The optimal hydrolysis time was previously determined as follows. EPS were weighed and diluted in the same volume of trifluoroacetic acid. Hydrolysis was interrupted after 10, 30, 50, 70, 90, 110, 150, 250, and 350 min and sugars quantified enzymatically for each microorganism and each type of monomer.

### 3.3. Monosaccharide Analysis with HPAEC

High-performance anion-exchange chromatography with pulsed amperometric detector (HPAEC-PAD) was used to identify and quantify the monosaccharides obtained after polysaccharide hydrolysis. The system used was ICS 3000 (Dionex Corporation, Sunnyvale, CA, USA). It consisted of an AS40 automatic injector, a pump module that can be operated in gradient mode, and a pulsed amperometric detector (PAD). Data acquisition and processing were done using Chromeleon software (version 6.8, ThermoFisher scientific, Waltham, Massachusetts, États-Unis).

The samples were filtered at 0.22 µm, and 25 µL of the samples was injected and then separated through a CarboPac PA1 pre-column and column (Dionex, 4 × 50 mm and 4 × 250 mm, respectively) and thermostated at 25 °C. The monosaccharides were transformed into alcoholates in the mobile phase at a pH above the pKa of their hydroxyl functions. The samples were eluted isocratically with 18 mM NaOH for 25 min, followed by a linear gradient between 0 and 0.5 M sodium acetate in 200 mM NaOH for 20 min, to elute the acidic monosaccharides. The run was followed by 15 min washing with 200 mM NaOH. The eluent flow rate was kept constant at 1 mL·min^−1^.

External standards (concentrations ranging from 0.001 to 0.01 g·L^−1^) were injected prior to each injection sequence (fucose, arabinose, galactose, glucose, rhamnose, xylose, mannose, fructose, ribose, galactosamine, glucosamine, *N*-acetylglucosamine, *N*-acetylgalactosamine, galacturonic acid, glucuronic acid).

### 3.4. Enzymatic Quantification of Free Sugar Monomers

In this work, the polysaccharides were synthetized by three microalgae and their compositions enzymatically characterized. The limitation of enzymatic kits is that they require previous knowledge of the polysaccharide composition in order to choose appropriate kit(s). The polysaccharide composition of the microalgae was found in the literature, and their monomeric sugar composition was determined using the reference chromatographic method (HPAEC-PAD). Each simple sugar was quantified with the following enzymatic kits (Megazyme) (the nature of the enzyme or the combination of enzymes used for the quantification is described in parentheses):

- l-fucose K-FUCOSE 02/17 (L-fucose deshydrogenase)
- d-galactose/l-arabinose K-ARGA 04/17 (galactose mutarotase + β-galactose deshydrogenase)
- d-glucose K-GLUHK-110A/K-GLUHK-220A 07/14 (hexokinase, glucose-6-phosphate dehydrogenase)
- l-rhamnose K-RHAMNOSE 02/15 (L-rhamnose dehydrogenase)
- Glucuronic/Galacturonic acids K-URONIC 04/16 (uronate dehydrogenase)
- d-xylose K-XYLOSE 04/16 (xylose mutarotase, β-xylose dehydrogenase)

These kits comprised buffers, coenzymes (NAD^+^ and NADP^+^), and one or two enzymes (as described above in parentheses). The targeted sugars accounted for more than 90% of the composition of the polysaccharides under analysis.

After acid hydrolysis and neutralization, the simple sugars were quantified following the protocols developed by Megazyme. Each quantification required 10 µL of hydrolysate. The volumes of buffer, coenzymes, and enzymes depended on the kit used. The final volume in each well was between 242 and 282 µL. Quantification was done by reading the absorbance at 340 nm on a Perkin Elmer EnSpire Multimode Plate reader with a Greiner microplate (96 wells).

### 3.5. Determination of Sensitivity

Limits of detection, quantification, and saturation (LOD, LOQ, and LOS, respectively) were determined for each EPS assay since the value evolves depending on the material used. To determine these parameters, simple sugar standards were prepared (from 8.62 × 10^−5^ to 0.04 g·L^−1^) and absorbance (340 nm) was measured in quintuplicate. The noise of the signal was calculated by dividing the standard deviation by the mean of each quintuplicate. The *LOD* and *LOQ* were calculated using Equations (1) and (2) [26]:

*LOD* = 3*S_a_*/*b* (1)

*LOQ* = 10*S_a_*/*b* (2)

where *S_a_* is the standard deviation for low concentration and *b* is the slope of the calibration curve.

The LOS was determined by the graphical method, assuming that the value corresponded to a point at which an increase in the standard concentration no longer caused an increase in absorbance.

### 3.6. Evaluation of Media Interferences on the Enzymatic Quantification Efficiency

Three kinds of media interference in efficient enzymatic quantification were evaluated: (i) the impact of the hydrolysis solution (mixture of TFA and NaOH), (ii) the presence of Amadori compounds produced by the Maillard reaction during the hydrolysis step, and (iii) the presence of salts from culture media.

After the hydrolysis step, the monomers were in solution in a mixture of TFA and NaOH (1 M each). To verify the effectiveness of the enzymes under these conditions, a mixture of monomers artificially reproducing the polysaccharide composition of *Pavlova* sp. (weight ratios: galactose (24.0%), glucose (20.7%), fucose (1.0%), rhamnose (36.0%), uronic acids (7.1%), xylose (10.9%)) was prepared in a solution of TFA and NaOH (1 M each). Each monomer was then enzymatically quantified to estimate the recovery rate.

The presence of proteins in purified EPS samples may cause a Maillard reaction during hydrolysis, leading to the formation of Amadori compounds. To check whether there was any interference with the enzymes during the quantification process, the standard addition method was employed. Five microliters of glucose at 2 g·L^−1^ was added to 245 µL of hydrolysate, and the glucose was then quantified (before and after supplementation) to confirm that the specificity of the enzyme was good enough to quantify glucose without interfering with the Amadori compounds.

Most chromatographic methods require a dialysis or purification step before quantifying the targeted sugars due to the presence of salts or other compounds that could cause a noisy response [3,16]. The sensitivity of the enzymes to the culture medium composition was estimated by quantifying a PS resolubilized in milli-Q water and BBM prior to hydrolysis. For this purpose, 148 µL of TFA (density 1.535 g.mL^−1^) was diluted in 852 µL of sample (PS diluted in milli-Q water or BBM) and then heated at 120 °C for 110 min.

### 3.7. Statistical Analysis

The variability of the method was tested by measuring each test in quintuplicate.

For comparison with the HPAEC method, the EPS from the three microorganisms studied were divided into 5 different samples (3 samples for the polysaccharide produced by *Synechoccocus* sp.) and then hydrolyzed separately in order to match the variability related to the hydrolysis protocol with the variability of the method. The data obtained were then subjected to a Kruskal-Wallis test to detect statistical differences between the results from the two methods.

The percentage of each monomer in a given polysaccharide (for a given hydrolysis) was calculated as follow:

(3) $$\%\;\mathrm{Galactose}=\frac{\sum \left[Galactose\right]}{TC_{a}\times n_{a}}$$

where *n_a_* and *TC_a_* represent the number of replicates used per measurement and the sum of the monomer concentrations calculated on polymer *a*, respectively.

The comparative study with the HPAEC method included hydrolysis variability, which led to the following calculation:

(4) $$\%\;\mathrm{Galactose}=\frac{\sum \frac{\sum \left[Galactose\right]}{TC_{a,b,c\ldots }\times n_{na,b,c\ldots }}}{n_{n}}$$

where *n_a,b,c,etc._* represents the replicate number used for hydrolysis (*a, b, c*, etc.), *n_n_* is the hydrolysate number studied, and *TC_n_* is the sum of each monomer concentration calculated per hydrolysate.

Dispersion was calculated by dividing the standard deviation by the mean of the percentage determined (see above).

## 4. Conclusions

The aim of the work presented in this paper was to develop and validate a rapid and cost-effective method for monitoring the monosaccharide profile of the (exo)polysaccharides produced by microalgae of nutraceutical and pharmaceutical interest. The method needing prior knowledge of the major sugars’ profiles, and it has to be used in a complementary manner to a reference chromatographic approach. The developed method was successfully applied directly on microalgae cultivated in a photobioreactor and a sample pre-treatment complex environment without any further conditioning step, relying mainly on the enzymes’ high specificity and sensitivity. For targeting the monosaccharides commonly associated with nutraceutical or pharmaceutical activities, i.e., fucose, rhamnose, and glucuronic/galacturonic acids, the method provided very good identification performance and should be used carefully for the specific case of fucose, which needs experimental protocol adaptation because of its thermal sensitivity. The method was also implemented on a microplate system in order to propose a multiplexed, sample savior and cost-effective assay.

Our proof of concept opens the way to a systematic, fast, convenient sugar profiling of bioactive polysaccharide extracts obtained from microalgae strains. The sensitivity and specificity of the method make it suitable for very small volumes of biomass and so support its use in systematic high-throughput screening studies for the discovery of new bioactive polysaccharides and the optimization of their production.

## Figures and Tables

**Table 1:** Comparison of the LOQ measured for enzymatic assays and one of the reference methods, as proposed by the literature (GC-MS: [27]; GC-FID (1): [28]; GC-FID (2): [29]; LC-MS-MS + TLC: [30]; RP-HPLC (1): [31]; RP-HPLC (2): [32]; HPAEC-UV-VIS + borate-HPAEC: [33]).

Monosaccharide/LOQ (ng/Injection)	Glucose	Galactose	Glucuronic Acid	Rhamnose	Fucose	Xylose
**Enzymatic**	9.6	17	9.5	14.1	7	4.2
**GC-MS**	40	40	82	67	68	34
**GC-FID (1)**	25	25	30	25	25	25
**GC-FID (2)**	480	710				
**LC-MS-MS**	0.05	0.1			0.05	0.1
**RP-HPLC (1)**	0.54	0.62	0.97	0.93	0.73	0.67
**RP-HPLC (2)**		2.27				
**HPAEC-UV-VIS**	0.33	1.9	3.7			1.6
**Borate-HPAEC**	13.9	18.8				7.3
**TLC**	900	750		575	ND	ND
**LC-MS**	38	49		17	90	66

**Table 2:** Deviation of the enzymatic method from HPAEC-PAD.

Deviation from HPAEC	Glucose	Galactose	Fucose	UA	Xylose	Rhamnose	Whole Polymer
*Pavlova* sp.	+7%	+14%	+82%	−35%	−12%	+11%	17%
*Synechococcus* sp.	−12%	+16%	+14%				12%
*P. cruentum*	+12%	−22%		−28%	+20%		22%

UA: uronic acids.

**Table 3:** Global coefficient of variation (including variations obtained on technical (Table S1) and hydrolysis replicates).

	Method	Glucose	Galactose	Fucose	UA	Xylose	Rhamnose
*Pavlova* sp.	Enzymes	12%	14%	16%	22%	15%	12%
	HPAEC	10%	9%	7%	10%	11%	12%
*Synechococcus* sp.	Enzymes	27%	33%	28%			
	HPAEC	30%	30%	20%			
*P. cruentum*	Enzymes	9%	9%		12%	13%	
	HPAEC	5%	5%		5%	7%	

UA: uronic acids (measurement replicates: 5; hydrolysis replicates: 5 for *Porphyridium cruentum* and *Pavlova* sp. and 3 for *Synechococcus* sp.).

**Table 4:** Methods, strengths, and weaknesses based on rapidity, water consumption, accessibility, and sensitivity.

	Methods	Time (min)	Usability	Mili Q Water (mL)	Error med.	Variability
*Pavlova* sp.	Enzymes	150	Easy to use	10	17%	15%
	HPAEC	276 (736)	Complex equipment	276 (736)		10%
*Synechococcus* sp.	Enzymes	90	Easy to use	4.8	12%	30%
	HPAEC	276 (736)	Complex equipment	276 (736)		27%
*Porphyridium cruentum*	Enzymes	90	Easy to use	6.4	22%	11%
	HPAEC	276 (736)	Complex equipment	276 (736)		6%

For HPAEC, the time required is indicated without or with (within parentheses) the standard curve establishment. The error med. represents the difference between the result obtained with the HPAEC method. For a given polysaccharide, the variability is the average variation coefficient calculated for each monomer.

## Supplementary Materials

The following are available online at https://www.mdpi.com/1660-3397/19/2/101/s1: Table S1: Coefficient of variation of the technical replicates obtained by the HPAEC and enzymatic methods (results presented for each monosaccharide as the average of the coefficients of variation obtained for different hydrolysates).
