# Supplementary material for: A New, Quick, and Simple Protocol to Evaluate Microalgae Polysaccharide Composition
Source: Mar Drugs. 2021 Feb 10;19(2):101. doi: 10.3390/md19020101 (PMC7916578; doi:10.3390/md19020101)
Supplement: Supplementary file 1 [file marinedrugs-19-00101-s001.pdf]

**Table S1.** Coefficient of variation of the technical replicates obtained by the HPAEC and enzymatic methods (results presented for each monosaccharide as the average of the coefficients of variation obtained for different hydrolysates).

| | Methods | glucose | galactose | fucose | UA | xylose | rhamnose |
|---|---|---|---|---|---|---|---|
| *Pavlova* sp. | Enzymes | 7% | 7% | 13% | 8% | 16% | 6% |
| | HPAEC | 4% | 5% | 14% | 10% | 13% | 4% |
| *Synechococcus* sp. | Enzymes | 8% | 5% | 6% | | | |
| | HPAEC | 4% | 7% | 20% | | | |
| *Porphyridium cruentum* | Enzymes | 9% | 7% | | 8% | 10% | |
| | HPAEC | 6% | 3% | | 10% | 8% | |
